# Shikonin Suppresses NLRP3 and AIM2 Inflammasomes by Direct Inhibition of Caspase-1

**DOI:** 10.1371/journal.pone.0159826

**Published:** 2016-07-28

**Authors:** Jernej Zorman, Petra Sušjan, Iva Hafner-Bratkovič

**Affiliations:** 1 Department of Synthetic Biology and Immunology, National Institute of Chemistry, Ljubljana, Slovenia; 2 Graduate School of Biomedicine, University of Ljubljana, Ljubljana, 1000, Slovenia; 3 EN-FIST Centre of Excellence, Ljubljana, Slovenia; Universidade de Sao Paulo, BRAZIL

## Abstract

Shikonin is a highly lipophilic naphtoquinone found in the roots of *Lithospermum erythrorhizon* used for its pleiotropic effects in traditional Chinese medicine. Based on its reported antipyretic and anti-inflammatory properties, we investigated whether shikonin suppresses the activation of NLRP3 inflammasome. Inflammasomes are cytosolic protein complexes that serve as scaffolds for recruitment and activation of caspase-1, which, in turn, results in cleavage and secretion of proinflammatory cytokines IL-1β and IL-18. NLRP3 inflammasome activation involves two steps: priming, i.e. the activation of NF-κB pathway, and inflammasome assembly. While shikonin has previously been reported to suppress the priming step, we demonstrated that shikonin also inhibits the second step of inflammasome activation induced by soluble and particulate NLRP3 instigators in primed immortalized murine bone marrow-derived macrophages. Shikonin decreased NLRP3 inflammasome activation in response to nigericin more potently than acetylshikonin. Our results showed that shikonin also inhibits AIM2 inflammasome activation by double stranded DNA. Shikonin inhibited ASC speck formation and caspase-1 activation in murine macrophages and suppressed the activity of isolated caspase-1, demonstrating that it directly targets caspase-1. Complexing shikonin with β-lactoglobulin reduced its toxicity while preserving the inhibitory effect on NLRP3 inflammasome activation, suggesting that shikonin with improved bioavailability might be interesting for therapeutic applications in inflammasome-mediated conditions.

## Introduction

Shikonin is a naphthoquinone found in the roots of *Lithospermum erythrorhizon Siebold & Zucc*. and other plants of the Boraginaceae family. Shikonin and its derivatives are the main active ingredients of Zicao, a traditional Chinese medicine used for its antibacterial, antiviral, anti-inflammatory and antipyretic effects. The indications for the use of *L*. *erythrorhizon* roots include skin lesions, burns, dermatitis, eczema, bedsores, sore throat, macular eruptions and measles [1, 2]. In recent decades, shikonin and its derivatives attracted large interest for their tumor-suppressing effects [3] and were shown to act on multiple cellular targets including pyruvate kinase-M2 (PKM2), transcription factor NF-κB, cyclooxygenase-2, TNF-α and STAT3 as reviewed by Andujar and coworkers [4]. In mouse models of lethal endotoxemia, shikonin reduced serum levels of an inflammatory mediator high-mobility group box 1 (HMGB1) protein and protected mice from LPS-induced death [5]. In normal conditions, HMGB1 acts as a transcriptional regulator, however when secreted from activated immune cells it mediates various inflammatory responses. HMGB1 is also secreted upon inflammasome activation [6].

Inflammasomes are cytoplasmic protein complexes that sense and are activated in response to pathogen- and danger-associated molecular patterns. They serve as scaffolds for recruitment and autoproteolytic activation of caspase-1, which cleaves the inactive precursor cytokines pro-IL-1β and pro-IL-18 into proinflammatory cytokines IL-1β and IL-18. The most studied NLRP3 inflammasome is composed of the sensor NLRP3, the adaptor protein ASC and pro-caspase-1. It is activated by a plethora of different stimuli ranging from small molecules such as ATP and microbial pore-forming toxins [7, 8] to aggregates including uric acid [9] and cholesterol crystals [10], silica [11, 12], asbestos fibers [11], amyloid β aggregates [13] and prion protein fibrils [14]. Inflammasome activation leads to efficient defense against pathogens, while missense mutations in the NLRP3 protein-encoding gene (*Cias1)* cause autoinflammatory disorders cryopyrinopathies (i.e. cryopyrin-associated periodic syndromes, CAPS), where IL-1β is constitutively processed [15–17]. Inappropriate activation of NLRP3 inflammasome is also implicated in common diseases including Alzheimer’s disease [18, 19] and diabetes [20]. The rapidly growing evidence for excessive inflammasome activation in a variety of common diseases highlights the need for small molecule inhibitors of NLRP3 inflammasome.

While NLRP3 inflammasome is controlled on transcriptional and posttranslational levels and by several endogenous inhibitors and modulators as reviewed by Rathinam and coworkers [21], current therapy of CAPS is based on the broad blockade of IL-1β or IL-1R signaling. No anti-IL-18 treatment is currently approved. Natural compounds parthenolide [22], artemisinin [23], scropolioside B [24] and curcumin [25, 26] have been shown to inhibit IL-1β secretion via NLRP3 inflammasome activation. Recently, one of the ketone bodies, β-hydroxybutyrate, was shown to specifically inhibit NLRP3 inflammasome [27] and synthetic compound MCC950 demonstrated effective and specific NLRP3 inflammasome inhibition with promising results in the mouse model of CAPS [28].

NLRP3 inflammasome activation requires prior expression of pro-IL-1β and NLRP3, which is achieved through activation of NF-κB. Versatile anti-inflammatory effects of shikonin have previously been observed both in cell culture and animal studies. Shikonin was shown to reduce edema in TPA-induced mouse ear edema model and effectively inhibited COX-2 and iNOS, all of which was attributed to decreased activation of NF-κB [29]. Inhibition of NF-κB by shikonin [29, 30] or shikonin/alkannin mixture [31] was confirmed in macrophage cell line RAW 264.7 stimulated by LPS.

In the present study we showed that shikonin inhibits NLRP3 inflammasome activation by acting on both signals. Previous studies demonstrated that it effectively inhibits NF-κB activation (i.e. the priming signal). We show that shikonin also dampens the maturation of pro-IL-1β by NLRP3 inflammasome in response to soluble and particulate NLRP3 instigators. We further demonstrated that shikonin also inhibits AIM2 inflammasome and that its effect on inflammasomes can be ascribed to both its inhibition of ASC oligomerization as well as its direct inhibition of caspase-1. Our results show that in addition to its previously reported targets, the observed anti-inflammatory effects of shikonin might also originate from its action on inflammasomes and caspase-1.

## Materials and Methods

### Materials

Shikonin (>98%, HPLC) was purchased from Enzo, acetylshikonin (>95%, HPLC) was from Biopurify Phytochemicals. Cell culture media, FBS and other cell culture supplies were from GIBCO, Alum was from Thermo Scientific, ultrapure LPS from Escherichia Coli O111:B4, silica (SiO_2_), LyoVec polydA:dT, imiquimod were from Invivogen. Nigericin, ATP and all other chemicals if not specified otherwise were from Sigma. Ready-SET-go ELISA kits (eBioscience) were used for determination of mouse and human IL-1β. Concentration of mouse IL-18 was determined by IL-18 Platinum ELISA (eBioscience).

### Cell Cultures

Immortalized BMDMs from C57BL/6 mice described in [12] were a kind gift of K. A. Fitzgerald (University of Massachusetts Medical school, Worcester, MA, USA). Immortalized BMDMs were cultured in DMEM supplemented with 10% FBS. THP-1 cells (ECCAC 88081201) were cultured in RPMI 1640 supplemented with 10% FBS.

### mRNA Isolation and qPCR

The messenger RNA (mRNA) was isolated using the RNeasy Mini Kit (Qiagen), transcribed to cDNA with High Capacity cDNA Transcription Kit (Applied Biosystems). qPCR was performed using the LightCycler 480 SYBR Green I Master mix on LightCycler 480 instrument (Roche).

### XTT and LDH Assays

Cells were cultured and treated as in experiments for IL-1β ELISA. Inflammasome stimulation was done in DMEM without phenol red. For XTT assay, cells in negative control were subjected to 0.1% Triton X-100 for 15 min. After supernatants were collected for IL-1β quantification, cells were cultured in DMEM without phenol red. A solution of XTT and phenazine methosulphate was prepared in DMEM without phenol red and added to the cells. After 9 h, absorbance at 490 nm (ref. 650 nm) was measured using multiplate reader SinergyMx (Biotek).

For LDH assay, supernatants were analyzed for the presence of LDH activity. Sample was mixed with lactate in Tris buffer, pH 8.2, and the mixture of phenazine methosulphate, NAD and iodonitrotetrazolium chloride. Supernatant from 0.1% Triton X-100 treated cells was used as positive control. After 15–30 min, absorbance at 490 nm was measured using multiplate reader SinergyMx (Biotek). LDH release in % was calculated using the supernatant of untreated cells as negative control and Triton X-100 treated supernatant as 100% LDH release.

### ELISA Assays

All experiments were performed in serum-free DMEM. Cells were seeded at 1.5 x 10^5^ cells per well of 96 well plate and primed with ultra-pure LPS (100 ng/mL) overnight for IL-1β or 8 h for IL-18. The growth medium was removed and potential inhibitors were added 30 min before the addition of stimulators. Further, different activators in DMEM were added for 1 h (nigericin, ATP), 6 h (silica), 12 h (alum) or 24 h (imiquimod). The concentration of secreted IL-1β and IL-18 was measured by ELISA (e-Bioscience) according to manufacturer’s instructions.

### ASC Speck Formation by Immunofluorescence

Endogenous ASC was labelled as previously described [32]. Cells were seeded into μ-slides (Ibidi). Priming, shikonin addition and nigericin treatment were done as described above. After 45 min of nigericin treatment, cells were fixed with paraformaldehyde (4%, Electron Microscopy Sciences) for 15 min, permeabilized by 0.2% saponin, 1% BSA in PBS for 30 min. Further, cells were incubated with primary antibody against ASC (Biolegend) for 1 h at room temperature and after washing in secondary Alexa Fluor 488 goat anti-mouse IgG (Invitrogen) in permeabilization/ blocking solution. After washing, Prolong Diamond Antifade solution with DAPI (Invitrogen) was added to wells. A Leica TCS SP5 laser scanning microscope mounted on a Leica DMI 6000 CS inverted microscope (Leica Microsystems, Germany) with an HCX plan apo 63× (NA 1.4) oil immersion objective was used for imaging. A 405 nm laser line of 20 mW diode laser was used for DAPI excitation and emitted light was detected between 415 and 450 nm. A 488 nm laser line of 100 mW argon laser with 10% laser power was used for detection of Alexa 488 conjugate, where emitted light was detected between 500 and 600 nm. For acquisition and image processing, Leica LAS AF software was used.

### Western Blotting

Experiments were performed as for measuring IL-1β ELISA, with exception that stimulation was done in 24-well plate format. Methanol precipitation was used to precipitate proteins from cell culture media. Cells were washed twice with cold PBS and lysed. Protein concentration in the cell lysate was measured with BCA. Proteins were separated on 15% SDS-PAGE gels, blotted onto the nitrocellulose membrane (GE Healthcare) and detected with appropriate primary and secondary antibodies for detection of caspase-1 p10: M-20 (Santa Cruz) and for the detection of caspase-1 p20 (Casper-1, Adipogen) followed by HRP-conjugated goat polyclonals to rabbit IgG (Abcam). NLRP3 was detected using Cryo-2 (Adipogen) as primary antibody and goat anti-mouse HRP conjugated antibodies (Jackson ImmunoResearch). SuperSignal West Femto Chemiluminescent Substrate (Thermo Scientific) was used for detection of HRP-labeled bands.

### Caspase-1 Activation

Activation of caspase-1 was measured by flow cytometer Cyflow (Partec) using Fluorochrome Inhibitor of Caspase 1 kit (Immunochemistry Technologies). Briefly, cells were seeded at 1.5 x 10^5^ cells per well of 24-well plate and primed with ultra-pure LPS (500 ng/mL) for 2 h. Potential caspase-1 inhibitors were added 30 min before activation with 10 μM nigericin. After 30 min FLICA reagent was added and incubated for 30 min. On ice, cells were washed twice, detached in PBS and analyzed by flow cytometer Cyflow (Partec).

### Caspase-1 Activity Assay

Potential inhibitors of caspase-1 were analyzed using Caspase-1 Inhibitor Drug Screening Kit (Fluorimetric) from BioVision. Shikonin and positive inhibition control (Z-VAD-FMK) were prepared in PBS and applied to black 96-well fluorescence plate (Corning). Active caspase-1 was added, followed by caspase-1 substrate YVAD-AFC. After 45 min incubation at 37°C, fluorescence of samples was measured using SinergyMx plate reader (Biotek).

### IC50 Calculation

The data were fitted using the 4 parameter logistic (4PL) nonlinear regression model and IC50 was calculated as the concentration of the inhibitor yielding half-maximal IL-1β release.

### Statistical Analysis

Unpaired two-tailed t-test was used for pairwise comparison.

## Results and Discussion

### Shikonin Inhibits the Maturation of IL-1β and LDH Release Induced by Nigericin, an Activator of the NLRP3 Inflammasome

**Fig 1:**
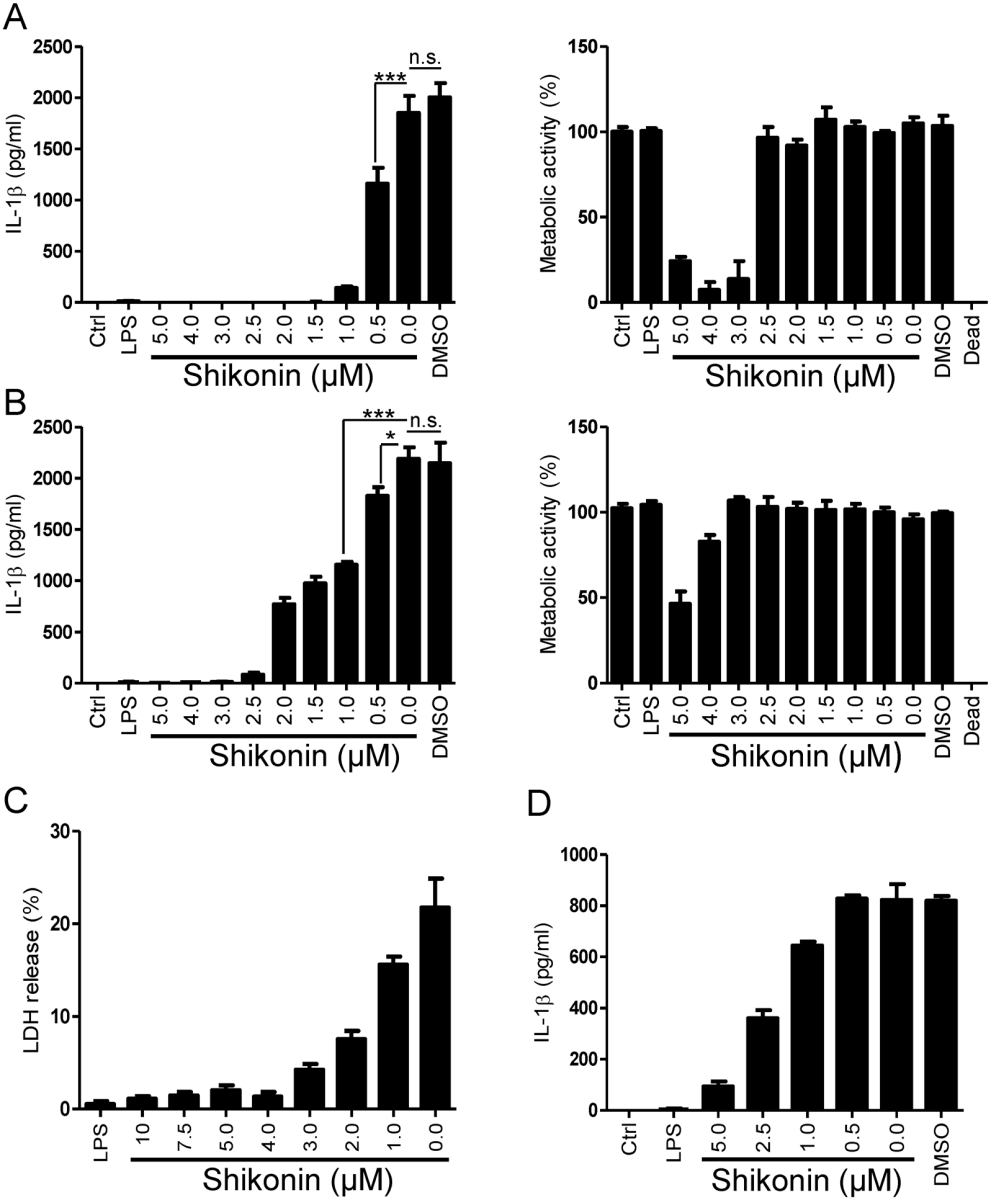
Shikonin Inhibits IL-1β Maturation and LDH release. (A, B) Shikonin inhibits IL-1β release from iBMDMs. Cells were primed with 100 ng/mL LPS for 12 h or left untreated (ctrl). Shikonin (5–0 μM) or vehicle (DMSO) was applied to cells 30 min before priming (A, left) or 30 min before activation with 10 μM nigericin (B, left) (nigericin is present in all samples but ctrl and LPS). Supernatants were collected 1 h after activation and assayed using IL-1β ELISA. * P ≤ 0.05, ** P ≤ 0.01, *** P ≤ 0.001. Metabolic activity of the remaining cells was determined with XTT assay (A right, B right). Cell death control was induced by 0.1% Triton X-100 (dead). (C) Shikonin inhibits the release of LDH into the culture medium of LPS-primed and nigericin-stimulated iBMDMs. (D) Shikonin inhibits human IL-1β release from PMA-differentiated and nigericin-stimulated THP-1 cells. THP-1 cells were differentiated into adherent macrophages with 500 ng/mL PMA overnight. Cells were activated as in (B). Representative of 3 (A–E) independent experiments is shown. Error bars represent SD of triplicate wells.

The activation of NLRP3 inflammasome is a two-step process. In the first, so called ‘priming step’, NLRP3 and pro-IL-1β are expressed upon activation of NF-κB. Since shikonin is a known NF-κB inhibitor [29, 30], it is expected that the priming step of inflammasome activation is affected. However, previously identified NF-κB inhibitors Bay 11–7082 and parthenolide were also shown to inhibit the second step of NLRP3 inflammasome activation [22]. We were interested whether shikonin was also able to inhibit IL-1β maturation. We followed the activation of inflammasome with nigericin in LPS-primed immortalized bone-marrow-derived macrophages from C57BL/6 mice (iBMDMs) [12]. When shikonin was added prior LPS stimulation, concentration-dependent inhibition of IL-1β maturation was observed (Fig 1A, left; IC_50_ range 0.3–0.6 μM). Interestingly, at slightly higher concentrations (IC_50_ range 1.4–2 μM), shikonin was also effective when added after the priming step, implying that it also inhibits the second step of NLRP3 inflammasome activation (Fig 1B, left). To support this finding, we also followed the effect of shikonin on transcription of pro-IL-1β by qPCR (S1 Fig). When shikonin was added prior to LPS, the expression of pro-IL-1β induced by LPS was reduced as expected since shikonin is known to suppress NF-κB activation (S1A Fig). However, when shikonin was added after LPS priming, there was no inhibition of pro-IL-1β expression (S1B Fig), demonstrating that the observed effect of shikonin on IL-1β maturation (Fig 1B) was not due to decreased expression of pro-IL-1β. Interestingly, the expression of NLRP3 on the protein level was unaffected by shikonin added either prior or after LPS stimulation (S1C Fig). Shikonin is able to kill tumor cells via either apoptosis (below 2.5 μM) or necroptosis (above 10 μM) [33]. As demonstrated by XTT proliferation assay, shikonin induced cell death, but at concentrations significantly higher than those required to inhibit IL-1β release (Fig 1A, right and Fig 1B, right), suggesting that inflammasome inhibition and induction of cell death are two separate effects of shikonin. Further, inhibition of IL-1β release was also achieved in the presence of Nec-1s, a necroptosis inhibitor (S2 Fig), which, however, did not inhibit cell death induced by shikonin in our experiments (not shown). One of the hallmarks of inflammasome activation includes necrotic cell death called pyroptosis. We showed that shikonin is able to dose-dependently inhibit LDH release induced by nigericin (Fig 1C). Shikonin also inhibited IL-18 release from LPS-primed mouse iBMDMs (S3 Fig). The concentration of shikonin required for this effect was higher than for inhibition of IL-1β maturation and approached cytotoxic levels of shikonin (Fig 1B, right). Furthermore, shikonin inhibited IL-1β release from PMA-differentiated and nigericin-stimulated human monocytic cell line THP-1 (Fig 1D). We demonstrated that shikonin at non-toxic concentrations inhibits NLRP3 inflammasome activation by nigericin.

### Shikonin is More Potent NLRP3 Inflammasome Inhibitor than Acetylshikonin

**Fig 2:**
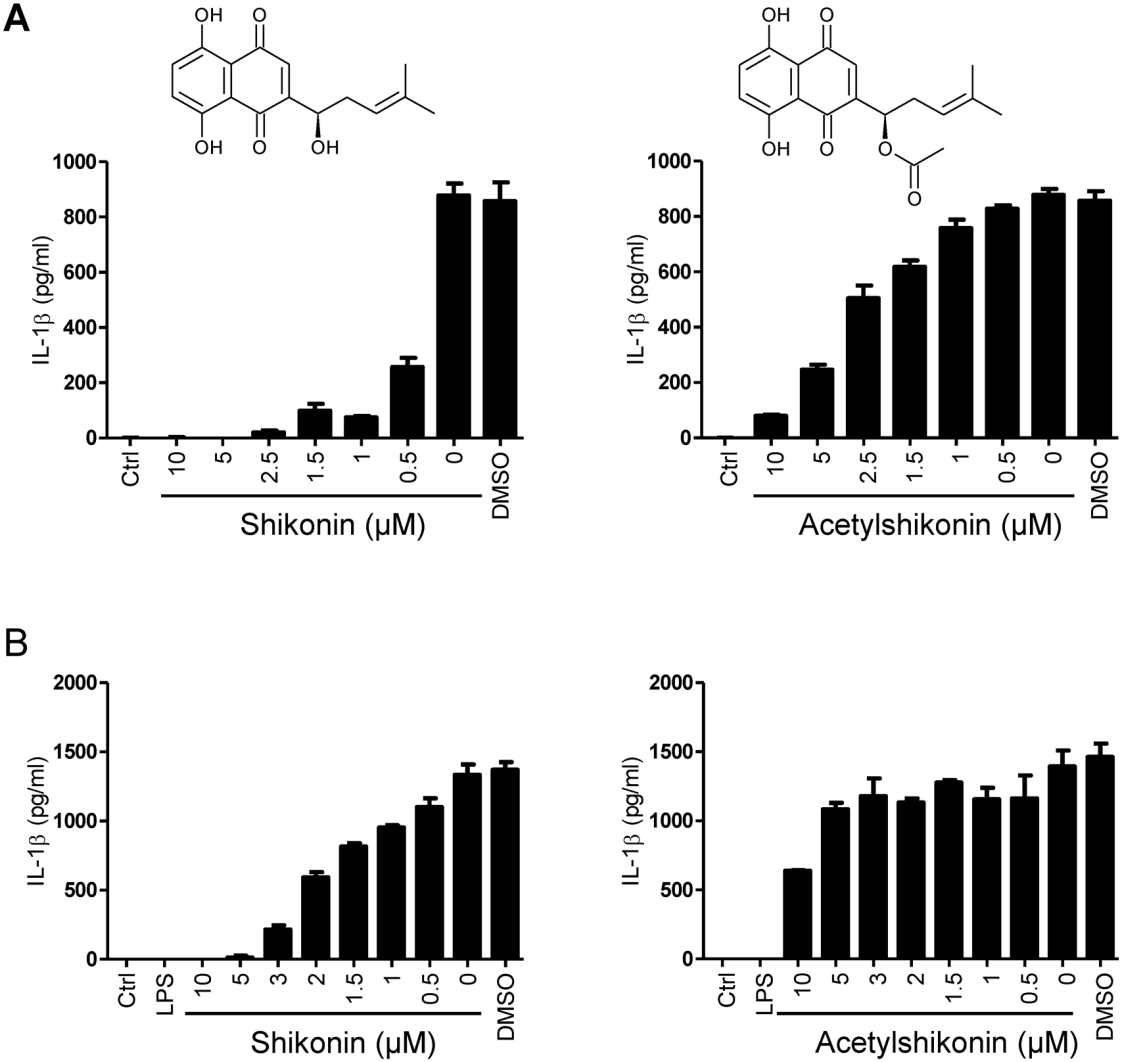
Shikonin is More Potent Inhibitor of IL-1β Maturation than Acetylshikonin. Shikonin (left) or acetylshikonin (right) was applied to cells 30 min before priming with 100 ng/mL LPS (A) or 30 min before activation with 10 μM nigericin (B). Supernatants were collected 1 h after activation with nigericin and assayed using IL-1β ELISA. Representative of three experiments is shown. Error bars represent SD of triplicate wells. Molecular structures of shikonin (left) and acetylshikonin (right) are shown above the corresponding results.

*L*. *erythrorhizon* root preparations contain several naphthoquinones, which were previously shown to exhibit anti-inflammatory effects as reviewed by Chen et al. [1]. Although the content of specific derivatives varies depending on the plant source and extraction procedure [1], acetylshikonin is more abundant in the roots of *L*. *erythrorhizon* than shikonin [1, 34]. We were interested whether acetylshikonin inhibits NLRP3 inflammasome similarly to shikonin. Shikonin and acetylshikonin were added either before priming (LPS) (Fig 2A) or before the addition of inflammasome instigator nigericin (Fig 2B, S4 Fig). Acetylshikonin concentration-dependently inhibited IL-1β release, particularly when added before the priming step. However, the inhibition achieved by acetylshikonin (IC_50_ ≅ 3 μM) was weaker compared to shikonin (IC_50_ range 0.3–0.6 μM). The difference was even more evident at the second step of NLRP3 inflammasome activation, where the inhibitory effects of shikonin and acetylshikonin had estimated IC50 values of 2 μM and 21 μM, respectively (Fig 2B left and S4 Fig). Previous studies demonstrated several anti-inflammatory effects of both compounds. Acetylshikonin was more potent inhibitor of neutrophil respiratory burst than shikonin [35]. On the other hand, Cheng and coworkers showed that shikonin/alkannin was superior to other derivatives including acetylshikonin in downregulating MAPK/NF-κB signaling [31], which agrees well with our results regarding the priming (NF-κB-dependent) step of inflammasome activation (Fig 2A). Our results show that shikonin is superior to acetylshikonin in suppression of the priming as well as the second step of NLRP3 inflammasome activation.

### Shikonin Inhibits Inflammasome Activation with Particulate and Soluble NLRP3 Instigators and by AIM2 Activator dAdT

**Fig 3:**
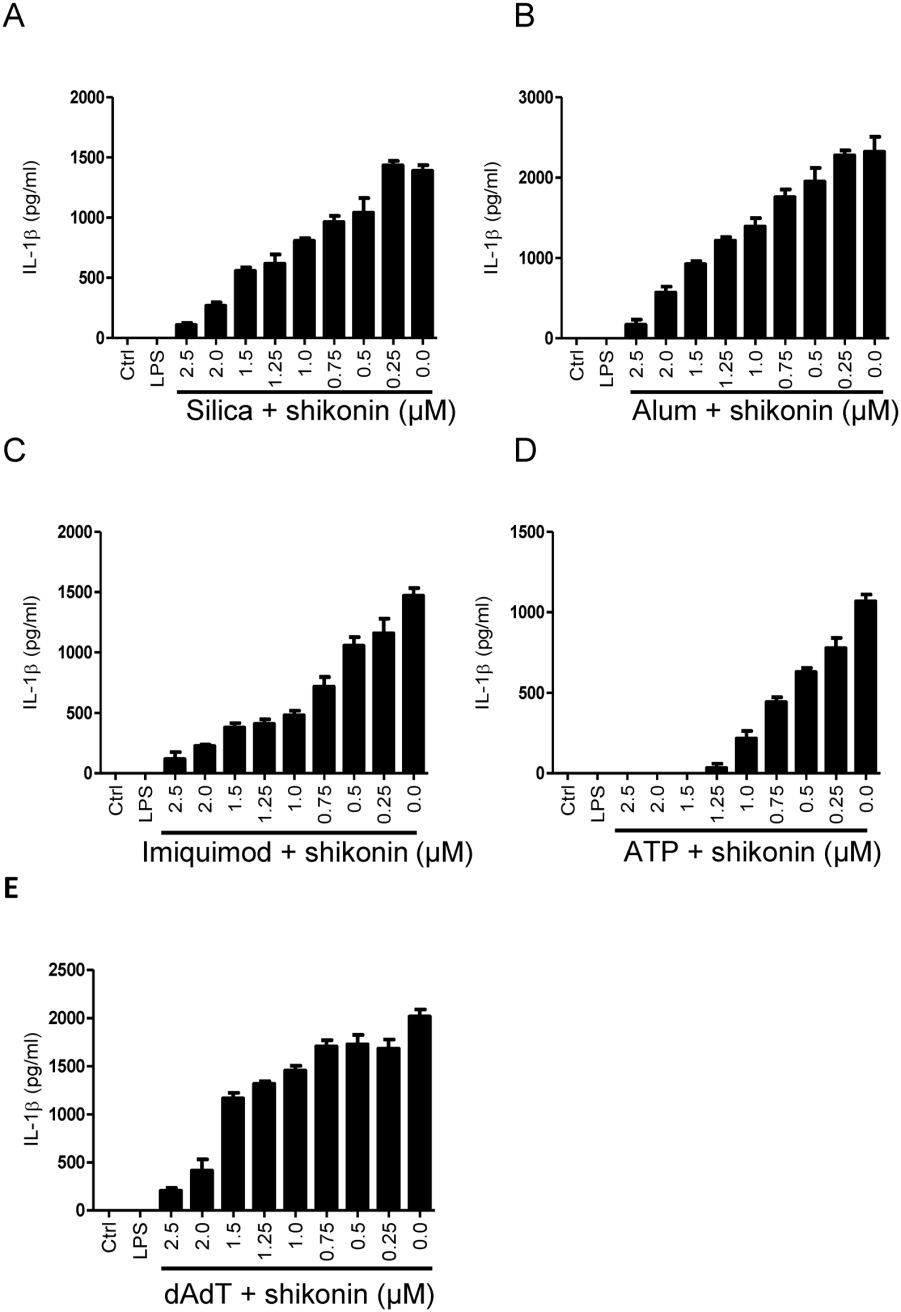
Shikonin Inhibits IL-1β Release from Macrophages Treated with Activators of NLRP3 Inflammasome and AIM2 Inflammasome. iBMDMs were primed with 100 ng/mL LPS for 12 h or left unprimed (ctrl). They were subjected to shikonin (2.5–0 μM) or DMSO (solvent control) 30 min before activation with 20 μg/mL silica (A) for 6 h, 0.5 mg/mL alum (B) for 12 h, 20 μg/mL imiquimod (C) for 24 h, 5 mM ATP (D) for 1 h or 1 μg/mL dAdT (E) for 1 h. Concentrations of mature IL-1β in culture media were determined using IL-1β ELISA. Representative of 3 (A to D) or 2 (E) independent experiments is shown. Error bars represent SD of triplicate wells.

NLRP3 inflammasome is unique in its activation by a wide variety of chemically and morphologically distinct stimuli. Several mechanisms of their action were proposed, for example decreased intracellular K^+^ [36, 37] or increased cytosolic Ca^2+^ [38–40]. In contrast to soluble activators, the action of particulate activators requires their phagocytosis and subsequent lysosomal destabilization [12]. To determine whether shikonin specifically inhibits NLRP3 inflammasome in response to nigericin or has broader action, we tested its inhibitory capacity in treatment with various NLRP3 inflammasome activators. While nigericin is a soluble toxin, we showed that shikonin also inhibits inflammasome activation with particulate triggers silica (Fig 3A) and alum (Fig 3B). Further, shikonin inhibited IL-1β secretion induced by soluble triggers imiquimod (Fig 3C) and ATP (Fig 3D). Our results demonstrate that shikonin inhibits NLRP3 inflammasome activation independently of the NLRP3 trigger used, suggesting that it acts at downstream stages of inflammasome activation. As the recruitment of adaptor protein ASC and activation of caspase-1 are common to several inflammasomes including the AIM2 inflammasome, we were interested whether shikonin specifically inhibits NLRP3 inflammasome. We showed that shikonin also inhibits IL-1β secretion induced by dAdT, an AIM2 inflammasome activator acting independently of NLRP3 (Fig 3E). Similar inflammasome inhibitory phenotype was previously observed for cysteinyl leukotriene receptor antagonist, which, however, had no effect on the priming step of inflammasome activation [41].

Our results demonstrated that shikonin interferes not with the action of different NLRP3 inflammasome instigators, but with the process of inflammasome assembly shared among several inflammasomes.

### Shikonin Inhibits Formation of ASC Specks and Directly Targets Caspase-1

**Fig 4:**
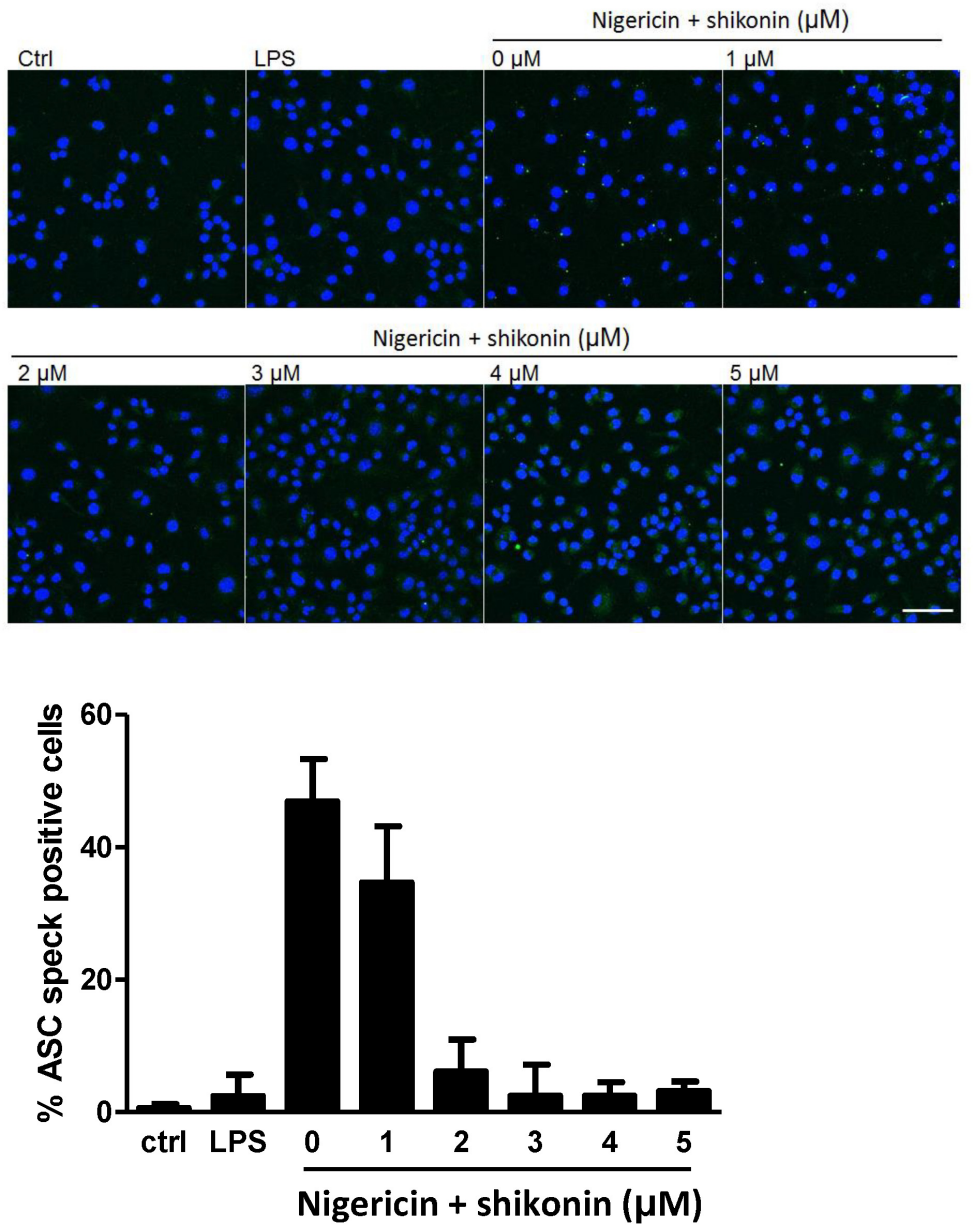
Shikonin Inhibits Formation of ASC Specks. iBMDMs were primed with 100 ng/mL LPS overnight or left untreated (ctrl). 30 min before stimulation with 10 μM nigericin iBMDMs were subjected to shikonin (0–5 μM). After 45 min, cells were fixed and labelled for endogenous ASC. At least five 250 μm x 250 μm fields were recorded for each condition and used for analysis (below). Bar represents 50 μm. Nuclei are depicted in blue (DAPI) and ASC in green.

Upon activation, both NLRP3 and AIM2 recruit the adaptor protein ASC, which multimerizes and acts as a platform for caspase-1 activation. Shikonin inhibited the activation of both NLRP3 and AIM2 inflammasomes suggesting that it either affects ASC oligomerization, pro-caspase-1 recruitment or its autoactivation. To determine whether shikonin affects ASC oligomerization, immunofluorescence of endogenous ASC was used [32]. In unstimulated or primed only cells, diffuse cytosolic stain was observed (Fig 4). Upon nigericin stimulation, however, ASC forms large oligomers called specks, resulting in condense bright spots in the perinuclear region. We showed that shikonin is capable of concentration—dependent inhibition of ASC speck formation (Fig 4).

**Fig 5:**
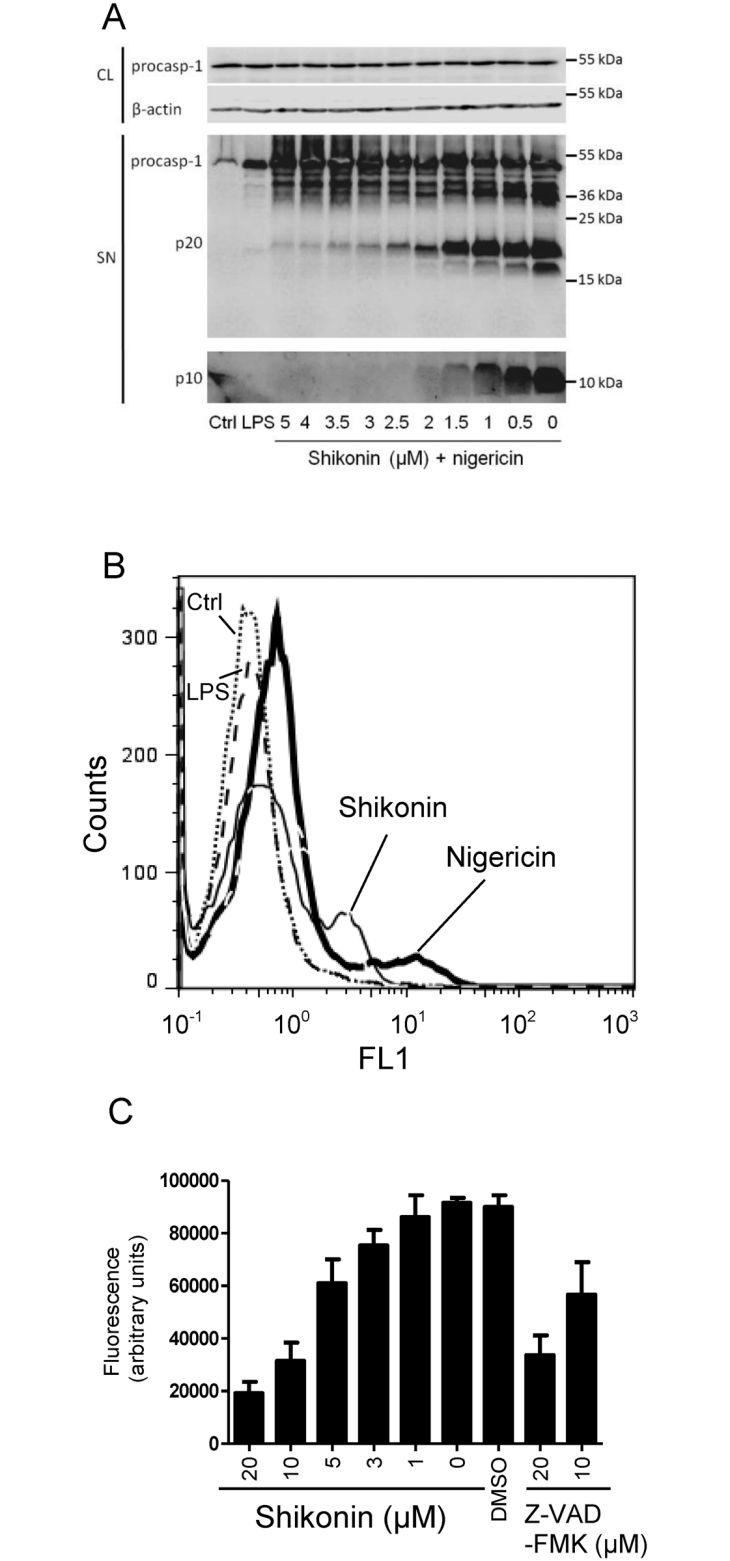
Shikonin Inhibits Caspase-1 Activation. (A) iBMDMs were primed with 100 ng/mL LPS overnight or were left untreated (ctrl). 30 min before stimulation with 10 μM nigericin, they were subjected to shikonin (5–0 μM) or solvent control (DMSO). Shikonin dose-dependently inhibited caspase-1 activation, which was followed by the presence of 20 kDa (upper blot) and 10 kDa (below) subunits of active caspase-1 in the supernatant. Pro-caspase-1 and β-actin in cell lysate are used as loading controls. Representative Western blot of 3 independent experiments is shown. (B) Activated caspase-1 in iBMDMs treated by nigericin (bold solid), nigericin and 0.5 μM shikonin (solid), LPS only (dashed) or untreated (dotted) was followed by flow cytometry upon binding of fluorescent FAM-YVAD-FMK. Representative of 2 independent experiments is shown. (C) Shikonin (20–0 μM) or DMSO (solvent control) and pancaspase inhibitor Y-VAD-FMK (20 and 10 μM) were tested for *in vitro* inhibition of caspase-1. Error bars represent SD of triplicate wells.

Further, we observed that shikonin concentration-dependently inhibited the secretion and processing of pro-caspase-1 from primed and nigericin-treated macrophages (Fig 5A). Decreased caspase-1 activation was also observed in shikonin-treated nigericin-stimulated murine macrophages using membrane-permeant fluorescent caspase-1 inhibitor as followed by flow cytometry (Fig 5B). While the effect on pro-caspase-1 and IL-1β processing could be the mere consequence of shikonin inhibiting ASC oligomerization, we were nevertheless interested whether shikonin has any effect on caspase-1 activity. In the *in vitro* assay using isolated caspase-1, we showed that shikonin directly inhibits caspase-1 in the same concentration range as the conventional caspase inhibitor Z-VAD-FMK (Fig 5C).

Previous studies showed that glutathione reacts with C2 position of naphthazarin (the ring skeleton of shikonin) [42] and shikonin was shown to react with cellular thiols [43]. It also reacted with the free thiol of cysteine residue in β-lactoglobulin, possibly via 1,4-reductive Michael addition [44]. As caspase-1 is a cysteine protease harboring cysteine in its active site, it is possible that shikonin reacts with this cysteine rendering the enzyme inactive. Collectively, our data demonstrated that shikonin concentration-dependently inhibits inflammasome activation by acting directly on caspase-1, however further studies are needed to elucidate the exact mechanism of shikonin inhibition of caspase-1 activity.

### Complexing Shikonin with β-lactoglobulin Decreases Its Cytotoxicity while Preserving the Inhibitory Capacity

**Fig 6:**
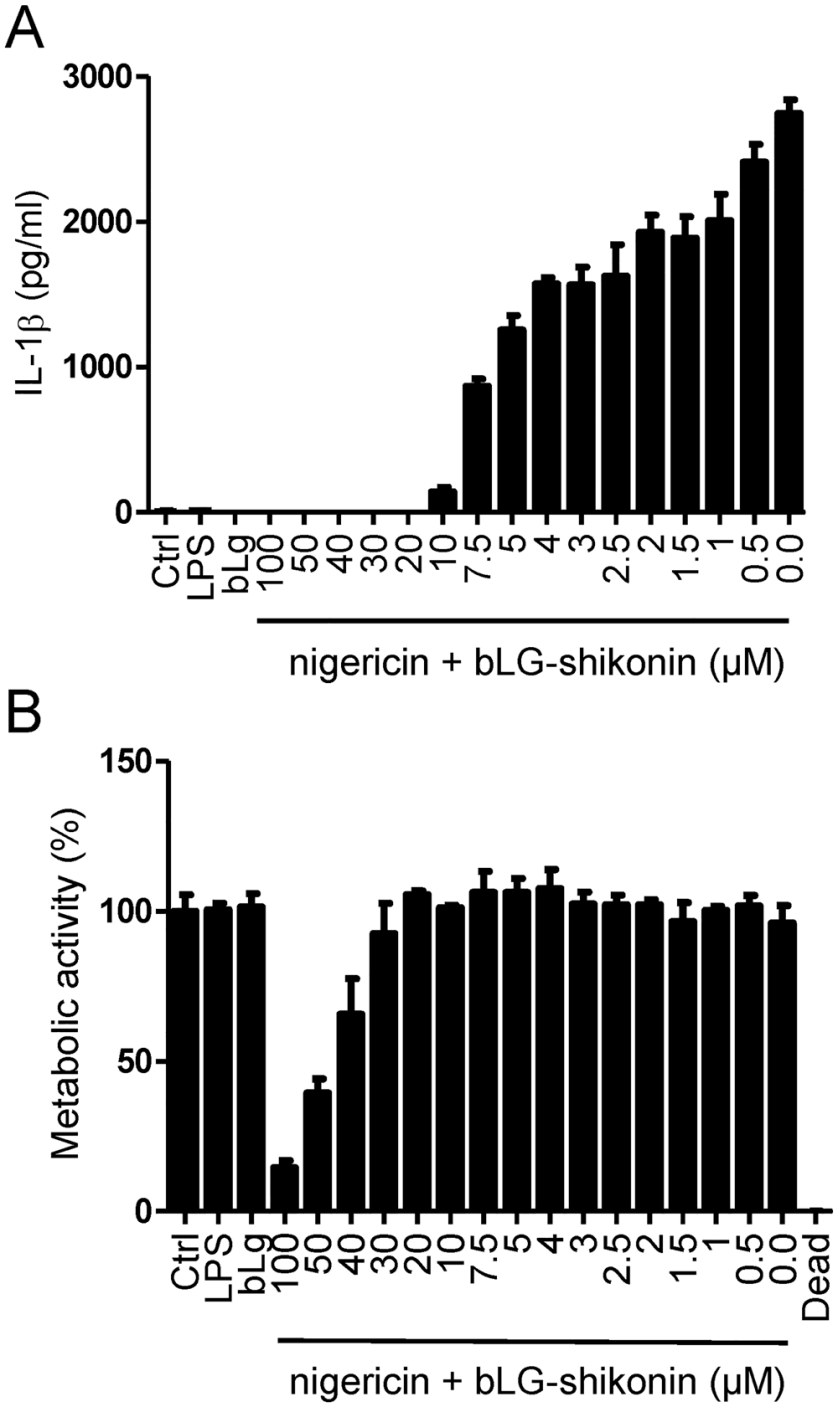
Complexing Shikonin with β-lactoglobulin Retains the Inhibitory Activity. iBMDMs were primed with 100 ng/mL LPS overnight or were left untreated (ctrl). The next day, they were activated with 10 μM nigericin. The complex of shikonin with β-lactoglobulin (molar ratio 1:100) (100–0 μM in shikonin) was applied to cells 30 min before activation with nigericin. (B) Cells exposed to samples of shikonin complexed with β-lactoglobulin showed conserved metabolic activity below 30 μM shikonin. Representative of 2 independent experiments is shown. Error bars represent SD of triplicate wells.

Shikonin is a highly lipophilic molecule. Since low aqueous solubility severely reduces its bioavailability, several approaches have been used to circumvent this problem. Increased aqueous solubility and decreased toxicity was, for example, achieved by complexing shikonin with hydroxypropyl-β-cyclodextrin [45] or β-lactoglobulin [44], encapsulation of shikonin in biocompatible materials [46, 47] or the use of biocompatible polymer electrospun fiber mats [48, 49]. We prepared a complex of shikonin with β-lactoglobulin as described by Albreht and coworkers [44]. β-lactoglobulin-complexed shikonin retained the ability to inhibit IL-1β maturation induced by nigericin, although higher concentrations of complexed shikonin were needed to achieve the same effect (Fig 6A). Importantly, complexed shikonin was less cytotoxic than free shikonin (Fig 6B). Decreased cell viability was observed at 30–40 μM β-lactoglobulin-complexed shikonin, while complete inhibition of NLRP3 inflammasome signaling was already evident at 10 μM. Our results corroborate the study of Xia and co-workers showing that shikonin-containing liposomes have lower toxicity compared to free shikonin [50] and encourage further studies in shikonin complexation aiming at reducing toxicity while preserving anti-inflammatory action.

With more than two millenia of use of shikonin-containing plant extracts in folk medicine, the molecular mechanisms underlying its effects were mostly discovered in the last three decades. In the present study, we corroborate previous reports documenting the inhibitory effect of shikonin on the NF-κB pathway. We further showed that shikonin suppresses NLRP3-dependent maturation of IL-1β induced by soluble as well as particulate triggers. How such diverse activators are able to induce the assembly of NLRP3 inflammasome is not known. One mechanism proposes the involvement of oxidative stress [51]. Shikonin was previously reported to be a reactive oxygen species (ROS) scavenger [52]. Recent studies, however, reported that shikonin is in fact a ROS producer that decreases tumor cell proliferation by ROS-induced apoptosis [53, 54], implying that inhibition of ROS is not the mechanism of action of shikonin on NLRP3 inflammasome. Shikonin was also reported to inhibit pyruvate kinase-M2 (PKM2) [55] thus reducing IL-1β and HMGB-1 release from LPS-challenged cells and protecting mice from lethal endotoxemia and sepsis [5]. Pyruvate kinase mediates the rate-limiting step of glycolysis. Aerobic glycolysis is important in tumor cells and in the activated immune cells [56]. Therefore, downregulation of PKM2 could account for the inhibitory action of shikonin on NLRP3 inflammasome. Our data, however, demonstrated that shikonin inhibits inflammasome activation by dampening the formation of ASC specks and by directly inhibiting caspase-1. The fact that the concentrations of shikonin required for caspase-1 inhibition in the *in vitro* assay were higher compared to cell culture-based systems supports the notion that shikonin inhibits inflammasome activation by acting on multiple targets. The emerging role of NLRP3 inflammasome and caspase-1 in diseases characterized by chronic inflammation (e.g. diabetes mellitus, Alzheimer’s disease, gout, multiple sclerosis, AIDS) provides the scope for development of novel approaches to previously incurable diseases. In this context, successful therapeutic implementation of small molecule inhibitors crucially relies on mechanistic understanding of their mode of action.

## Supporting Information

S1 Fig Effect of Shikonin on Expression of pro-IL-1β mRNA and NLRP3 Protein. Cells were primed with 100 ng/mL LPS for 12 h or left untreated (ctrl). Shikonin (1 μM) was applied to cells either 30 min before priming (A and C, before) or after priming for 30 min (B and C, after). (A, B) Two biological replicates were subjected to qPCR analysis to determine the relative abundances of pro-IL-1β mRNA, which are expressed as fold increase compared to the mRNA amounts in untreated cells. GAPDH was used as the reference and ΔΔCT method was used for quantification. * P ≤ 0.05, ** P ≤ 0.01, *** P ≤ 0.001. Representative of three independent experiments is shown. (C) Representative Western blot of two independent experiments is shown.
(PDF)

S2 Fig Necroptosis Inhibitor Nec-1s Does Not Affect Shikonin-Mediated Inhibition of IL-1β Release. iBMDM cells were primed with 100 ng/mL LPS overnight or left untreated (ctrl). (A) 2 μM Nec-1s was applied to cells 30 min before the addition of shikonin. (A, B) Shikonin (10–0 μM) or vehicle (DMSO) was applied to cells 30 min before activation with 10 μM nigericin (omitted in ctrl and LPS). Supernatants were collected 1 h after activation and assayed using IL-1β ELISA. Representative of two experiments is shown. Error bars represent SD of triplicate wells.
(PDF)

S3 Fig Relatively High Concentrations of Shikonin are needed for Inhibition of IL-18 release from iBMDMs. Cells were primed with 100 ng/mL LPS for 8 h or left untreated (ctrl). Shikonin (7.5–0 μM) or vehicle (DMSO) was applied to cells 30 min before activation with 10 μM nigericin (nigericin is present in all samples but ctrl and LPS). Supernatants were collected 1 h after activation and assayed using IL-1β ELISA. Representative of three experiments is shown. Error bars represent SD of triplicate wells.
(PDF)

S4 Fig High Concentrations of Acetylshikonin (>20 μM) Moderately Inhibit IL-1β Release. Acetylshikonin was applied to iBMDM cells 12 h after priming with 100 ng/mL LPS and 30 min before activation with 10 μM nigericin. Supernatants were collected 1 h after activation and analyzed by IL-1β ELISA. Representative of three experiments is shown. Error bars represent SD of triplicate wells.
(PDF)
