# Correction: Shikonin Suppresses NLRP3 and AIM2 Inflammasomes by Direct Inhibition of Caspase-1

**DOI:** 10.1371/journal.pone.0163887

**Published:** 2016-09-22

**Authors:** Jernej Zorman, Petra Sušjan, Iva Hafner-Bratkovič

The reference number 32 was incorrectly cited. The correct reference for the protocol for detection of ASC specks by immunofluorescence is: 32. Baroja-Mazo A, Martin-Sanchez F, Gomez AI, Martinez CM, Amores-Iniesta J, Compan V, et al. The NLRP3 inflammasome is released as a particulate danger signal that amplifies the inflammatory response. Nat Immunol. 2014;15(8):738–48. doi: 10.1038/ni.2919. PubMed PMID: 24952504.

## References

[1] Zorman J, Sušjan P, Hafner-Bratkovič I (2016) Shikonin Suppresses NLRP3 and AIM2 Inflammasomes by Direct Inhibition of Caspase-1. PLoS ONE 11(7): e0159826 doi:10.1371/journal.pone.0159826 27467658 10.1371/journal.pone.0159826 PMC4965082

