# Supplementary figures and images for: Shikonin Suppresses NLRP3 and AIM2 Inflammasomes by Direct Inhibition of Caspase-1
Source: PLoS One. 2016 Jul 28;11(7):e0159826. doi: 10.1371/journal.pone.0159826 (PMC4965082; doi:10.1371/journal.pone.0159826)

A

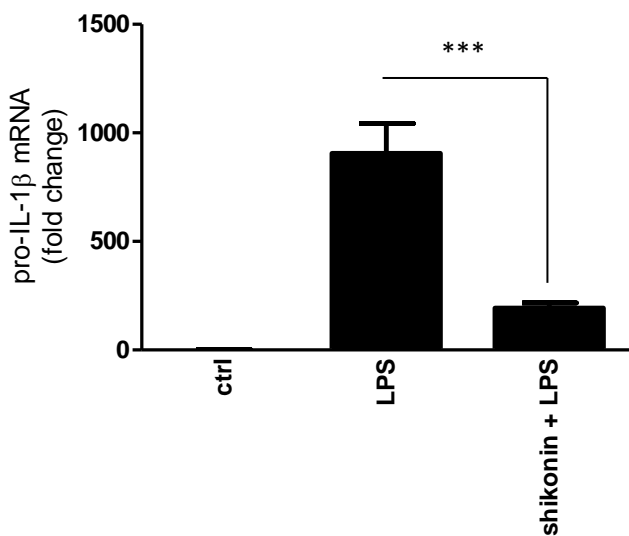

B

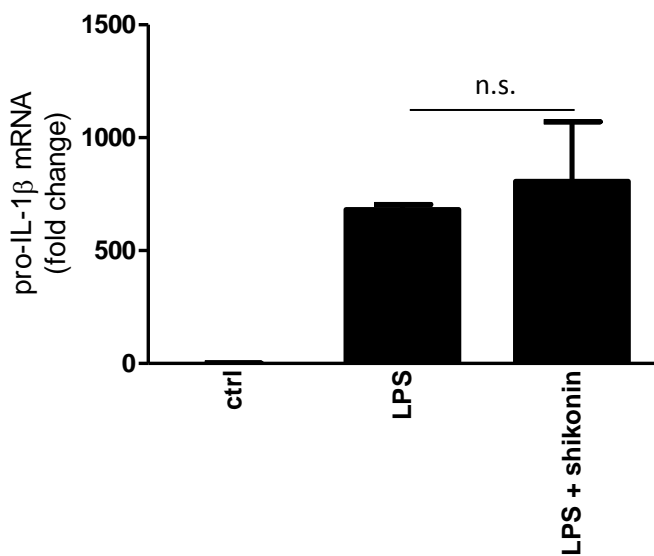

C

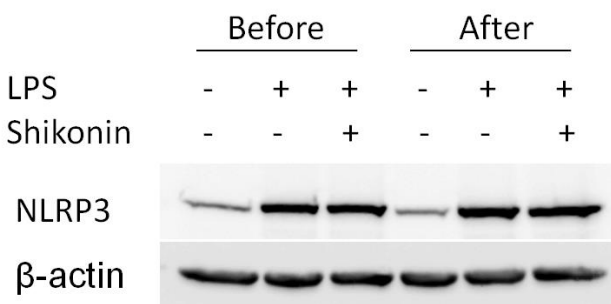

Supplement: S1 Fig — Cells were primed with 100 ng/mL LPS for 12 h or left untreated (ctrl). Shikonin (1 μM) was applied to cells either 30 min before priming (A and C, before) or after priming for 30 min (B and C, after). (A, B) Two biological replicates were subjected to qPCR analysis to determine the relative abundances of pro-IL-1β mRNA, which are expressed as fold increase compared to the mRNA amounts in untreated cells. GAPDH was used as the reference and ΔΔCT method was used for quantification. * P ≤ 0.05, ** P ≤ 0.01, *** P ≤ 0.001. Representative of three independent experiments is shown. (C) Representative Western blot of two independent experiments is shown. (PDF) [file pone.0159826.s001.pdf]

**A**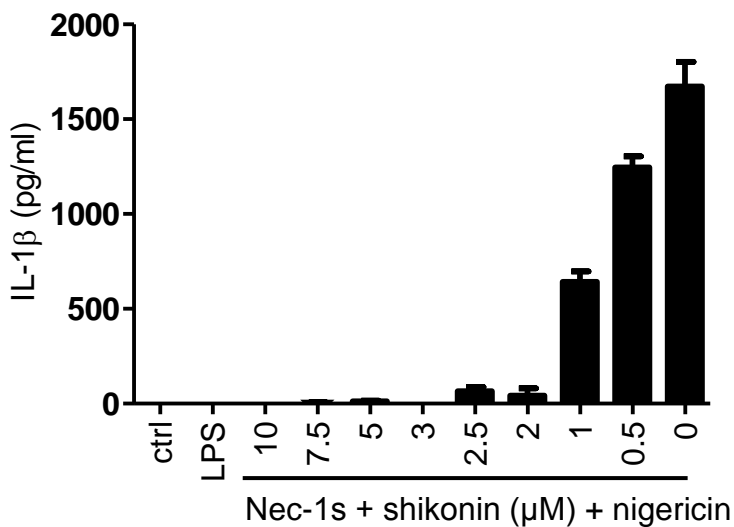**B**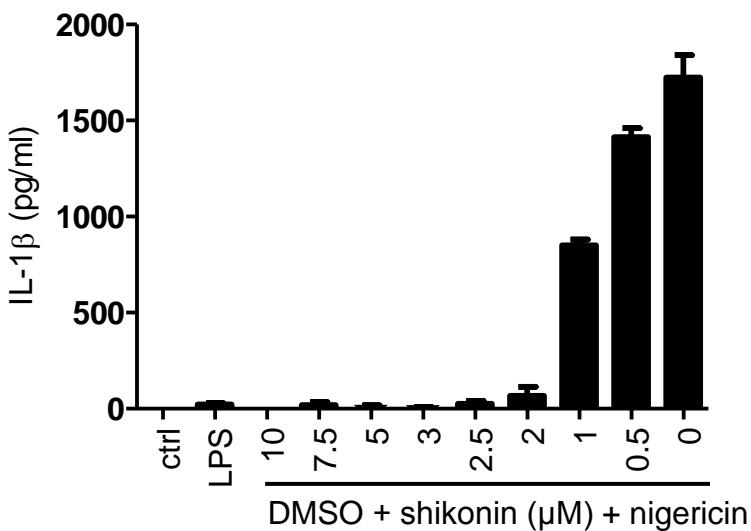

Supplement: S2 Fig — iBMDM cells were primed with 100 ng/mL LPS overnight or left untreated (ctrl). (A) 2 μM Nec-1s was applied to cells 30 min before the addition of shikonin. (A, B) Shikonin (10–0 μM) or vehicle (DMSO) was applied to cells 30 min before activation with 10 μM nigericin (omitted in ctrl and LPS). Supernatants were collected 1 h after activation and assayed using IL-1β ELISA. Representative of two experiments is shown. Error bars represent SD of triplicate wells. (PDF) [file pone.0159826.s002.pdf]

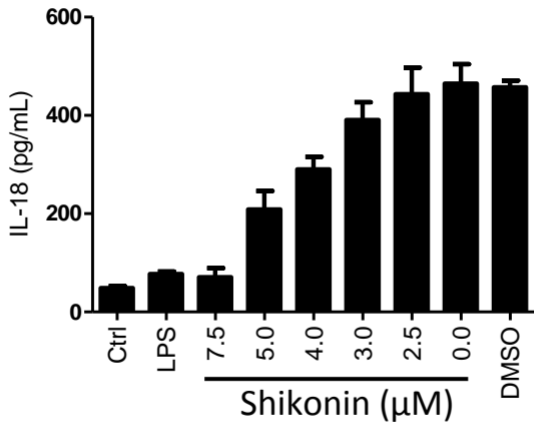

Supplement: S3 Fig — Cells were primed with 100 ng/mL LPS for 8 h or left untreated (ctrl). Shikonin (7.5–0 μM) or vehicle (DMSO) was applied to cells 30 min before activation with 10 μM nigericin (nigericin is present in all samples but ctrl and LPS). Supernatants were collected 1 h after activation and assayed using IL-1β ELISA. Representative of three experiments is shown. Error bars represent SD of triplicate wells. (PDF) [file pone.0159826.s003.pdf]

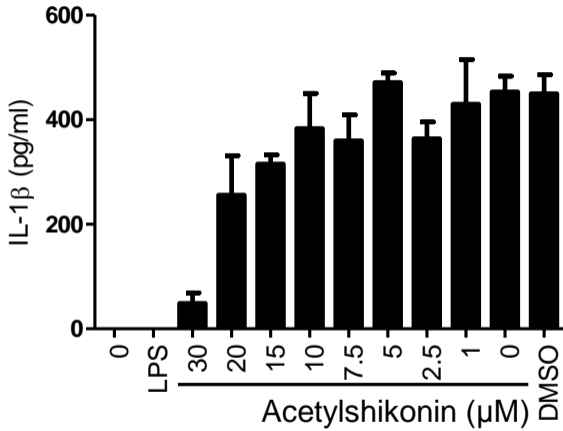

Supplement: S4 Fig — Acetylshikonin was applied to iBMDM cells 12 h after priming with 100 ng/mL LPS and 30 min before activation with 10 μM nigericin. Supernatants were collected 1 h after activation and analyzed by IL-1β ELISA. Representative of three experiments is shown. Error bars represent SD of triplicate wells. (PDF) [file pone.0159826.s004.pdf]
